# Radiobiology of the C3H Mouse Mammary Carcinoma: Increased Radiosensitivity In Situ of Homoplasts attenuated Prior to Implantation

**DOI:** 10.1038/bjc.1954.32

**Published:** 1954-06

**Authors:** A. Cohen, L. Cohen


### RADIOBIOLOGY OF THE C3H MOUSE MAMMARY CARCINOMA: INCREASED RADIOSENSITIVITY *IN SITU* OF HOMOPLASTS ATTENUATED PRIOR TO IMPLANTATION.

A. COHEN AND L. COHEN.

*From the Experimental Oncology Laboratory, Radiation Therapy Department, Johannesburg General Hospital.*

Received for publication February 11, 1954.

THERE are many reported examples of animals having been rendered immune or resistant, by various procedures, to subsequent inoculations of a tumour to which they are normally susceptible. The attenuation of tumour fragments by irradiation before implantation will, on occasion, produce antigenic changes in the tumour sufficient to induce immunity. Goldfeder (1942) first pointed out the quantitative relationship between the attenuation dose and the immunising property of the implant, in this case Sarcoma 180. With an adequately inbred strain like the C3H mouse, however, and using homologous mammary tumour which has not lost its genetic specificity by repeated serial transplantation, absolute resistance to further implants of the same tumour cannot be evoked.

It was previously shown that the radiosensitivity of a homologously-transmitted tumour is a function of the state of resistance of the host. When the resistance of a tumour-bearing C3H mouse is impaired by total body irradiation, the effective dose for treatment of the tumour increases; and, conversely, when some degree of resistance in a host arises from a relative genetic incompatibility of the host-tumour relationship, as in the case of hybrids bearing the parent strain tumour, the required dose is significantly decreased (Oughterson, Tennant and Lawrence, 1940). In this respect, estimation of an induced increase in radiosensitivity may be a sensitive indicator of subliminal host resistance when other criteria are lacking.

The significance of this concept was emphasised when " takes " arising from tumour fragments irradiated prior to implantation in the course of a previous experiment (Cohen and Cohen, 1953*a*) were subsequently treated and found to have a significantly increased radiosensitivity. It seemed probable that this change in radiosensitivity was a result of a minor degree of induced resistance in these animals, and a further investigation was undertaken to elucidate the respective rôles of the host and the tumour in this phenomenon.

### EXPERIMENT 1.

### *Materials and methods.*

This preliminary investigation was based on 51 mice which had been inoculated with tumour fragments, irradiated in a plastic welled slide immediately before implanatation with doses of 2000 or 2500 r, as described in our first communication (Cohen and Cohen, 1953*a*). Practically all implants attenuated at these sublethal doses " took," after a more or less prolonged latent period averaging 30 days, and then grew progressively at the normal growth rate. When the tumours had reached the standard size of about 1 cm. in the largest diameter (overall average volume 250 $\pm$ 81 mm.$^3$), they were irradiated *in situ* with our standardised technique as previously reported in detail. All mice were individually numbered and examined at regular intervals for at least 3 months after treatment. With the doses used in this series, the tumour has, within this observation period, either disappeared completely or has resumed active growth, ulcerating the skin and killing the mouse by exsanguination or infection.

In order to detect any acquired increase in radiosensitivity, tumours arising from each attenuation-dosage group were treated at three dose levels : 3500, 4200 and 5000 r, making a total of six categories tested. Since the probability of curing an unattenuated C3H tumour in the control series with 4200 r is less than 0·1 per cent, even a few cures at this dose in an experimental series would be significant. The proportion of cures obtained at each level was tabulated and analysed by the probit method, estimating the median effective dose ($LD_{50}$), the slope and linearity of the regression line, and the magnitude and significance of the difference between these results and the previously established parameters for the control series.

*Results.*

The proportion of cures obtained in each of the six experimental categories is shown in Table I. It is fairly obvious, and readily confirmed by analysis of the data, that for any given dose *in situ* there is no significant difference between the response of tumours attenuated at 2000 r and at 2500 r. Accordingly, the pooled results from both attenuation groups, given various doses *in situ*, are shown in Table I and plotted in Fig 1 (Line G) where they may be compared with the

FIG. 1.—Probit diagram showing response to treatment at various doses of homoplasts of the C3H carcinoma attenuated before implantation (Line G), compared with previously established data for the unattenuated tumour (Line A). Also shown are the proportion of cures when attenuated homoplasts are transplanted to other animals (Points H, J) ; transplanted to another site in the donor mice (Point I); or recurrent in the site of surgical excision (Point K).

control series (Line A). It will be noted that the probit regression line in the attenuated series differs both in position and slope from that of the controls.

The $LD_{50}$ for the attenuated homoplasts is found to be 4500 ($\pm$ 200*) r, compared with 5700 r previously determined in the controls, and the coefficient of variation is 26 per cent compared to 9 per cent in the controls. The *relative radiosensitivity* of attenuated tumours with reference to the controls, that is the ratio of the $LD_{50}$'s is $\frac{5700}{4500} = 1{\cdot}27$ ($\pm$ 0·07*). Since this ratio exceeds unity by four times its standard error, the result is highly significant ($p < 0{\cdot}0001$), indicating a definite acquired radiosensitivity of the tumour arising from an attenuated implant.

As has been previously shown, a changed radiosensitivity generally reflects an underlying change in host-resistance to the tumour. It would appear, therefore, that the attenuation procedure, while not producing any absolute immunity to further implants of the same tumour, nevertheless induces within the host, albeit the strain of origin of the tumour, a subliminal resistant state. The increased slope of the regression line compared with the control series cannot be explained by sporadic fluctuations in cure-rates, but implies a significant individual variation, presumably reflecting some irregular reaction or an inconstant degree of resistance arising in the C3H host in response to attenuated tumour fragments.

TABLE I.—*Response to Treatment* in situ *of Homoplasts (C3H Mammary Tumour) Attenuated by Irradiation Prior to Implantation.*

| | Attenuation dose. | | | | | |
|---|---|---|---|---|---|---|
| | 2000 r | | 2500 r | | | |
| Dose *in situ* (r). | Number of mice | Number cured. | Number of mice. | Number cured. | Total cures. | Cures (per cent). |
| 5000 | 5 | 3 | 13 | 8 | 11/18 | 61 |
| 4200 | 12 | 7 | 7 | 3 | 10/19 | 52 |
| 3500 | 6 | 0 | 8 | 0 | 0/14 | 0 |

### EXPERIMENT 2.

For the purpose of discerning whether the acquired radiosensitivity of attenuated homoplasts was (*a*) a persistent measurable change in the attenuated "take," (*b*) a local effect at the site of implantation or, (*c*) a manifestation of systemic resistance in the host, a second experiment was done.

### *Materials and methods.*

Using the same mouse strain and tumours as in the first experiment, the following procedure was adopted. Tumour fragments were irradiated in the plastic slide described previously with doses of 2000 r, and then inoculated subcutaneously into the right flanks of 11 mice. Although the latent period was increased to an average of 27 days, the growth rates of the established tumours were not less than that of unirradiated implants. Each tumour was allowed to grow to a diameter of approximately 1 cm., and then excised with a sterile surgical technique and fragments

* Standard errors of median and of ratios of medians.

re-inoculated into the left flank of the tumour-bearer, and, at the same time into 2 other mice (recipients). All inoculations " took " (reverting to the normal latent interval), and there were also recurrences in 7 of the 11 surgical donor sites, giving 40 tumours in all.

Using the irradiation procedure previously described, all " donors " were treated with 4200 r *in situ*, bilaterally where necessary. Of the 22 " recipients " (2 to each donor), 11 were similarly treated *in situ* with 4200 r in order to detect persistent acquired radiosensitivity in the tumour transplants, and the remaining 11 were treated with 7500 r *in situ* to exclude the possibility of an acquired radioresistance. The former dose will result in practically no cures of unattenuated C3H mammary tumour homoplasts, so that even a few cures in the first group would be statistically significant ; while with the latter dose the probability of cure exceeds 99·8 per cent, so that a few failures would indicate a significant acquired radioresistance.

*Results.*

At the beginning of this series of treatments, an immediate radiation mortality was noted in donors in which bilateral tumours had been treated on the same day. Since a follow-up of about 3 months after treatment is essential for evaluating cures, it was subsequently attempted to minimise the lethal effect by more careful surgical eradication of the original tumour, thus obviating the necessity for bilateral irradiation, and allowing a period of recovery of at least a week between treatments when bilateral tumours still occurred. The added disadvantage of bilateral treatments, even when the immediate mortality could be reduced, was the possibility that the increased body dose would lower the expected percentage of cures, as previously demonstrated (Cohen and Cohen, 1953*b*). The results of treatment of the attenuated homoplasts in the four experimental categories are shown in Table II, and the proportion of cures in each group plotted in Fig. 1 for comparison with the other relevant data.

TABLE II.—*Response to Treatment* in situ *of Attenuated Homoplasts after Excision and Transplantation.*

| | Symbol (Fig. 1). | Dose *in situ* (r). | Number of mice. | Number cured. | Cures (per cent). | Normally expected cure rate (per cent). |
|---|---|---|---|---|---|---|
| Donors: | | | | | | |
| Recurrent in site of excision | K | 4200 | 7 | 0 | 0 | 0·1 |
| Transplanted in opposite side | I | 4200 | 11 | 4 | 37 | 0·1 |
| Recipients I | H | 4200 | 11 | 0 | 0 | 0·1 |
| Recipients II | J | 7500 | 11 | 11 | 100 | 99·8 |

In spite of the adverse effects of bilateral irradiation, it can be seen in Table II that a significant proportion of cures (37 per cent) did eventuate in the case of the *re-inoculated* tumours in the donors treated with 4200 r. From Fig. 1 it can be seen that the proportion of cures to be expected at this dosage with unattenuated homoplasts (Line A) is exceedingly small, while in animals bearing attenuated implants (Line G) it is 40 per cent, from which this result (Point I) does not differ appreciably. On the other hand, the combination of body dose and damage of the vascular stroma by surgery may be considered responsible for the sharp reduction in cure-rate of the tumour recurrences in the excision sites (0/7 cures), even where increased radiosensitivity of the tumour on the other side existed. These findings eliminate the possibility that the increased radiosensitivity is due to a local resistance arising during the sojourn of the attenuated homoplast at the original implantation site, but indicate the existence of a generalised or systemic change.

Reference to Table II also shows that, when the attenuated "take" was transplanted to other recipients, the resultant tumours were neither more radiosensitive (0/11 cures at 4200 r), nor less radiosensitive (11/11 cures at 7500 r) than the unattenuated control homoplast in the C3H mouse (Cohen and Cohen, 1953*a*). In this connection it is interesting to note that, although the original tumour fragments attenuated with 2000 r prior to implantation exhibited the prolonged latent interval of 27 days, the latent period of the subsequent transplants of these "takes," both in the donors and in the new recipients, was the same as unattenuated tumour (7 to 15 days). These observations indicate that, in so far as its growth rate and radiosensitivity in new hosts are concerned, the previously attenuated homoplast has apparently recovered to a large extent from the initial irradiation.

### DISCUSSION.

The most significant finding in these experiments is that some degree of resistance arises in a proportion of C3H mice as a result of the inoculation of a viable mammary tumour fragment, attenuated with a known sublethal dose of X-rays prior to implantation. Aside from the prolonged latent period before such an attenuated implant becomes palpable (Goldfeder, 1947), no other gross evidence of alteration as a result of such a procedure has ever been previously reported for this type of host and tumour. This subliminal resistance, or change in the host-tumour relationship, is manifested by an increase in the radiosensitivity of the attenuated "take." It is of interest to note that the response of radiation-attenuated homoplasts in the C3H mouse closely resembles, both in the magnitude of the effective dose and in the individual variance, that of unattenuated C3H tumour growing in factor-harbouring (C3H $\times$ CBA) $F_1$ hybrids (Cohen and Cohen, 1954). The attenuation procedure, therefore, seems to induce a degree of resistance, presumably a similar antigenic difference between host and tumour, at least as great as that engendered by the tumour growing in $F_1$ hybrids harbouring the milk factor.

The following facts concerning the nature of this phenomenon have been elucidated :

(1) While not excluding the possibility that the tumour cells have been inherently changed by this procedure, it is obvious that any such change is not, in itself, sufficient to affect the radiosensitivity of the transplanted attenuated "takes" in the environment of a new host. Conversely, there is no evidence of acquired radioresistance in the tumour as a result of previous irradiation, in conformity with Montgomery and Warren's findings (1953).

(2) It is unlikely that any local change in the tumour bed contributes to the changed radiosensitivity since no cures were obtained when recurrences following the local excision at the original site were treated. It can probably be assumed, too, that damage to the tumour bed by the surgical excision was sufficient to abrogate the effect of systemic resistance.

(3) Since the increased radiosensitivity persists even where the tumour has been transplanted to another site in the original donor, it must be concluded that the effect is due to a systemic, generalised resistance mechanism.

The phenomenon of induced resistance to experimentally transmitted cancer has been extensively investigated. In the past, results in this field were often contradictory and confusing (Woglom, 1929), due to the use of heterogeneous strains of animals and gentically alien tumours. Under these circumstances, absolute immunity to tumours can be readily induced by a variety of experimental procedures. There are also reported instances of absolute immunity to a transplanted tumour being induced in the strain of origin, even where the animals are genetically homogeneous (Gross, 1943 ; Goldfeder, 1945 ; Lewis and Aptekman, 1952 ; Foley, 1952). In each of these reports, however, the tumour had been repeatedly subpassaged for many generations before its immunising ability was tested. The long transplantation history of these and other commonly used tumours, maintained solely by serial transmission, is surveyed by Dunham and Stewart (1953). It is generally realised that such procedures may permit antigenic diversification or mutation in the tumour, which, in effect, results in a relatively unstable host-tumour relationship. Consequently, many of these findings are largely invalidated in so far as their applicability to human cancer therapy is concerned.

It would appear that only when a supply of spontaneous tumours is constantly available within a given strain, and is routinely transplanted through no more than a few subpassages to experimental animals of the same strain, can the host-tumour relationship be considered adequately stabilised. Where such stringent conditions prevail, absolute immunity to a tumour cannot be evoked (Bittner, 1936 ; Barrett, 1940 ; Lewis, 1940 ; Fardon and Prince, 1953 ; Foley, 1953). A recent review by Hauschka (1952) contains an extensive analysis of the immunogenetic complexities of host-tumour relationships used in experimental cancer reseaı ch.

With respect to the C3H mammary carcinoma, the authors have shown that, even where some degree of immunogenetic difference existed between the tumour and the host, as in the case of $F_1$ hybrids bearing the parent strain tumour, no overt-immunity could be elicited by curing the comparatively radiosensitive tumour *in situ* (Cohen and Cohen, 1954), or by previously inoculating the animals with radiation-attenuated fragments (unpublished data).

When one correlates these facts with the findings of the foregoing experiment, it becomes evident that resistance to tumour is by no means an " all-or-none " phenomenon. It seems likely that partial or subliminal states of resistance can be induced in the stable host-tumour relationship which, while not obviously detectable by conventional methods, nevertheless yield significant results in the form of a perceptible increase in radiosensivity.

In this connection, it is significant that Gorer (1947) demonstrated high titres of circulating anti-tumour iso-antibodies in mice dying of foreign strain tumour transplants, which grew progressively in spite of antigenic differences with the hosts. This form of apparently ineffective resistance may be identical with that responsible for the enhanced response to radiotherapy, from which one might postulate that iso-antibodies may be elicited by the attenuation procedure even where obvious genetic differences between host and tumour do not exist. It is suggested that an efficient method of rendering homologous tumour antigenic to its host would have a potent radiosensitizing effect.

SUMMARY.

C3H mice bearing homoplasts of the C3H mammary adeno-carcinoma, which had been attenuated prior to implantation with 2000 r and 2500 r, were treated *in situ*. The $LD_{50}$ of these tumours was found to be 4500 ($\pm$ 200) r, which is significantly lower than that of unattenuated homoplasts (5700 r).

The increased radiosensitivity was not a local phenomenon, but persisted in the original hosts even where the established attenuated homoplast was excised and transplanted to another site. When the previously attenuated homoplasts were transplanted from the original hosts to new hosts, however, the curative dose reverted to that of unattenuated tumour.

It is considered probable that the inoculation of a viable tumour fragment, attenuated by sublethal doses of radiation prior to implantation, induces a state of subliminal resistance in the host which enhances the radiosensitivity of the resultant tumour.

Facilities for the maintenance of our experimental animals were generously provided at the South African Institute for Medical Research by Dr. J. F. Murray, to whom we are deeply indebted.

### REFERENCES.

BARRETT, M. K.—(1940) *J. nat. Cancer Inst.*, **1**, 387.
BITTNER, J. J.—(1936) *Amer. J. Cancer*, **28**, 121.
COHEN, A., AND COHEN, L.—(1953*a*) *Brit. J. Cancer*, **7**, 231.—(1953*b*) *Ibid.*, **7**, 452.—(1954) *Ibid.*, **8**, 303.
DUNHAM, L. J., AND STEWART, H. L.—(1953) *J. nat. Cancer Inst.*, **13**, 1299.
FARDON, J. C., AND PRINCE, J. E.—(1953) *Cancer Res.*, **13**, 9.
FOLEY, E. J.—(1952) *Proc. Soc. exp. Biol. N.Y.*, **80**, 675.—(1953) *Cancer Res.*, **13**, 578.
GOLDFEDER, A.—(1942) *Radiology*, **39**, 426.—(1945) *Proc. Soc. exp. Biol. N.Y.*, **59**, 104.—(1947) *Radiology*, **49**, 724.
GORER, P. A.—(1947) *Cancer Res.*, **7**, 634.
GROSS, L.—(1943) *Ibid.*, **3**, 326.
HAUSCHKA, T. S.—(1952) *Ibid.*, **12**, 615.
LEWIS, M. R.—(1940) *Johns Hopk. Hosp. Bull.*, **67**, 325.
*Idem* AND APTEKMAN, P. M.—(1952) *Cancer*, **5**, 411.
MONTGOMERY, P. O., AND WARREN, S.—(1953) *Radiology*, **60**, 421.
OUGHTERSON, A. W., TENNANT, R., AND LAWRENCE, E. A.—(1940) *Yale J. Biol. Med.*, **12**, 419.
WOGLOM, W. H.—(1929) *Cancer Rev.*, **4**, 129.


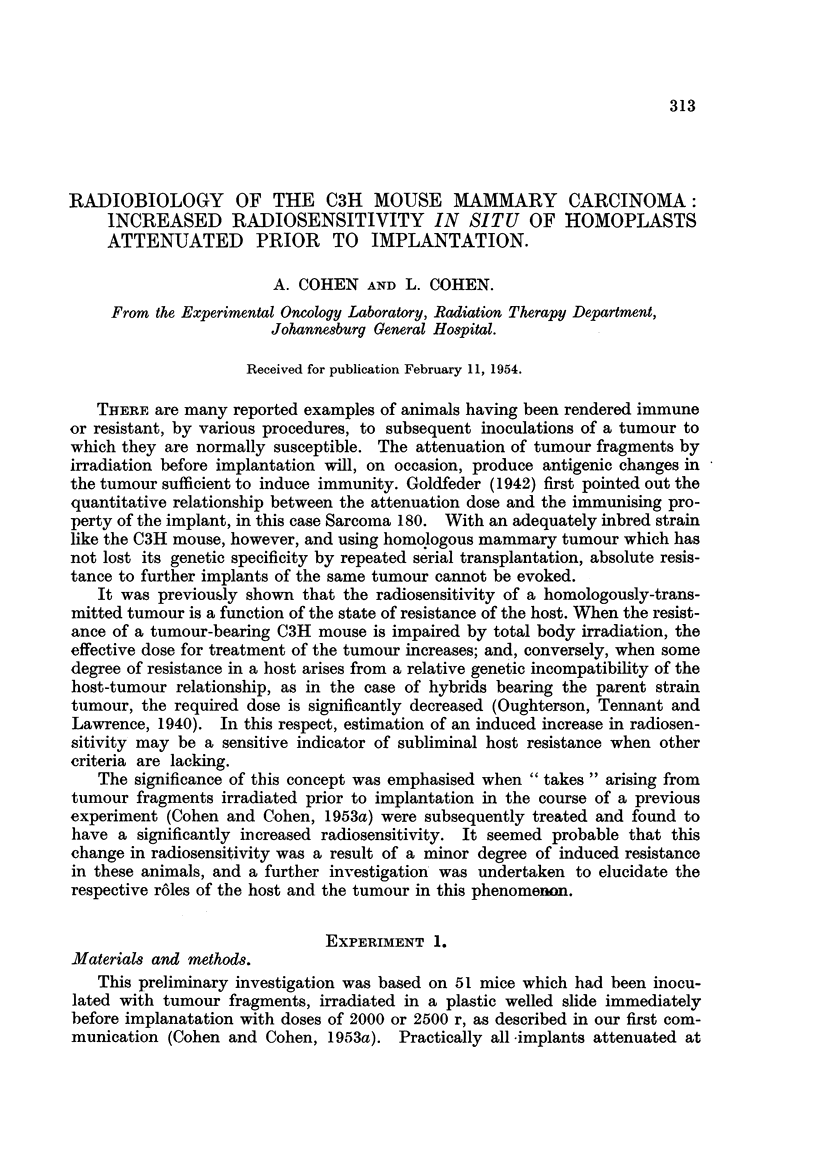

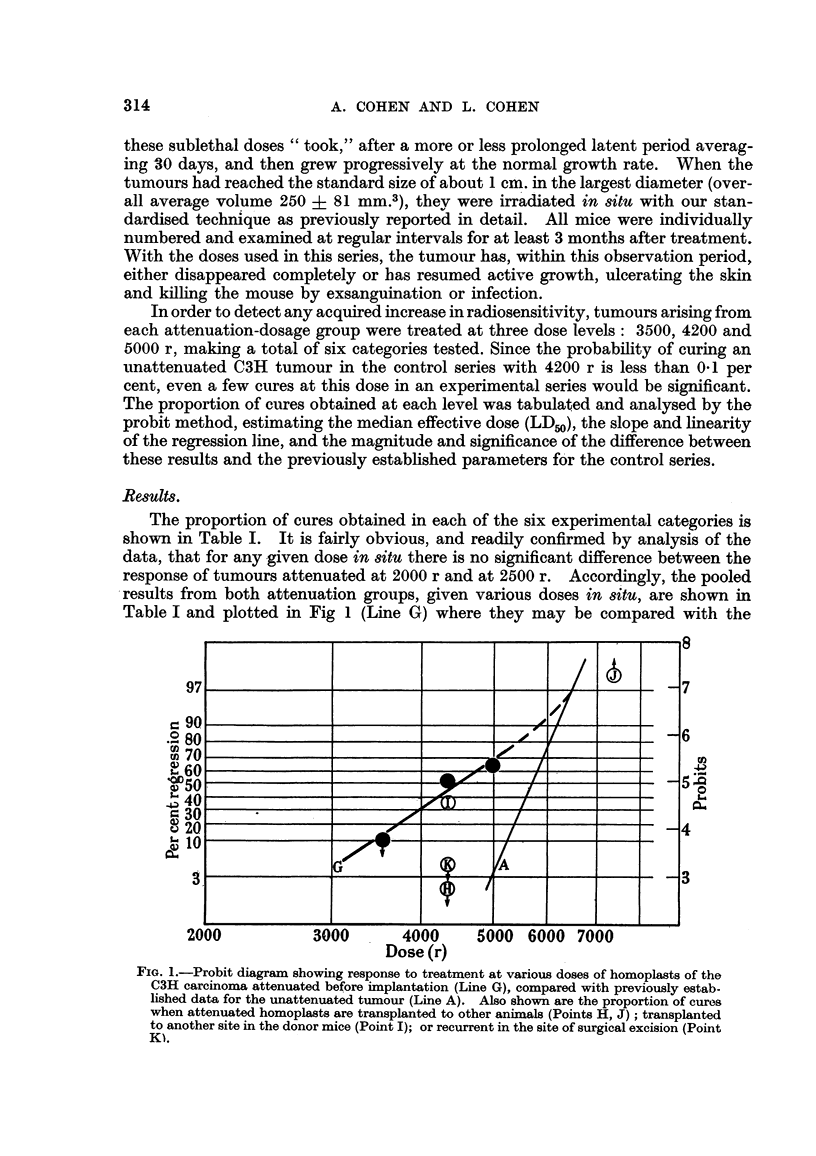

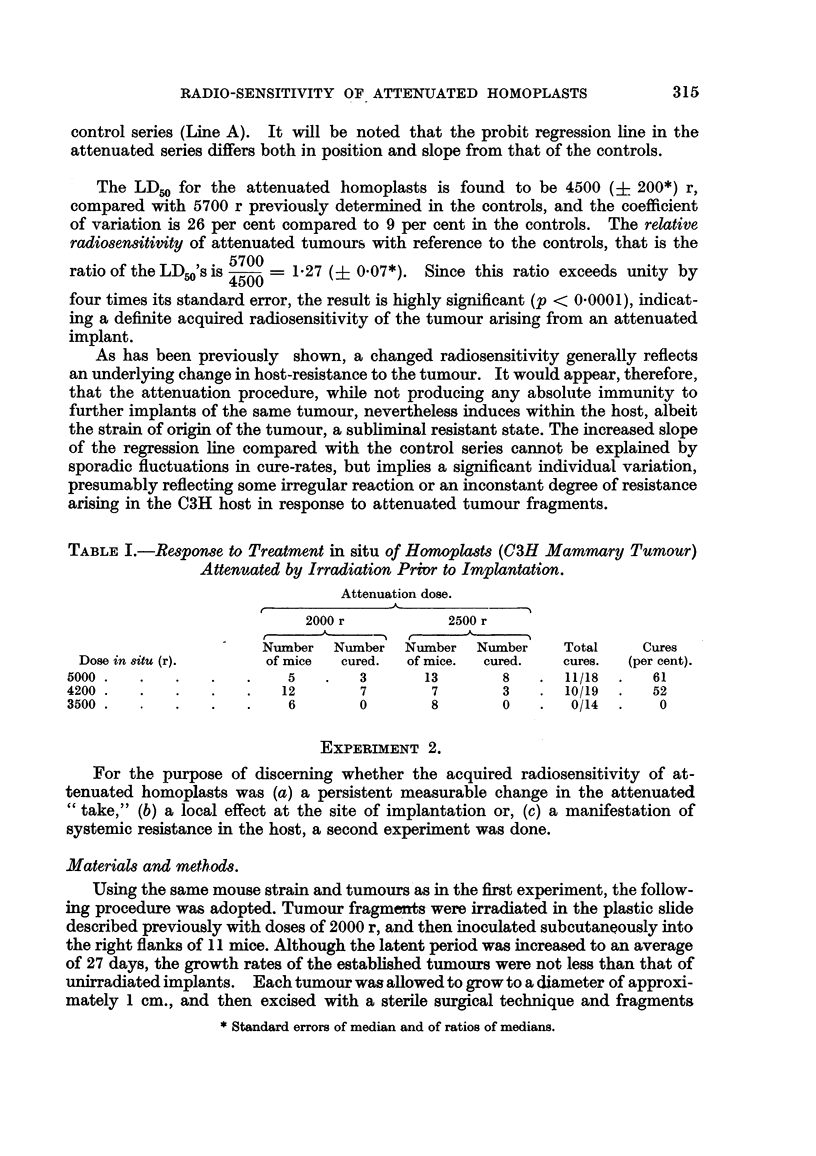

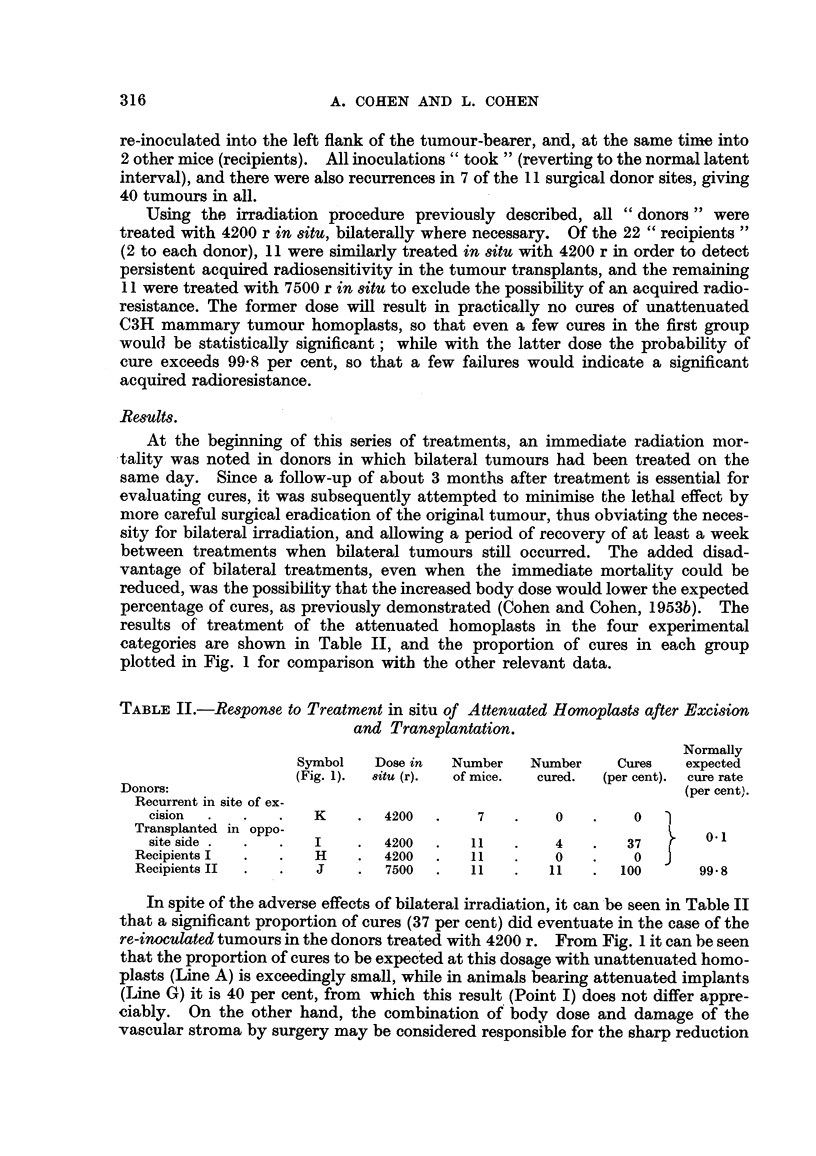

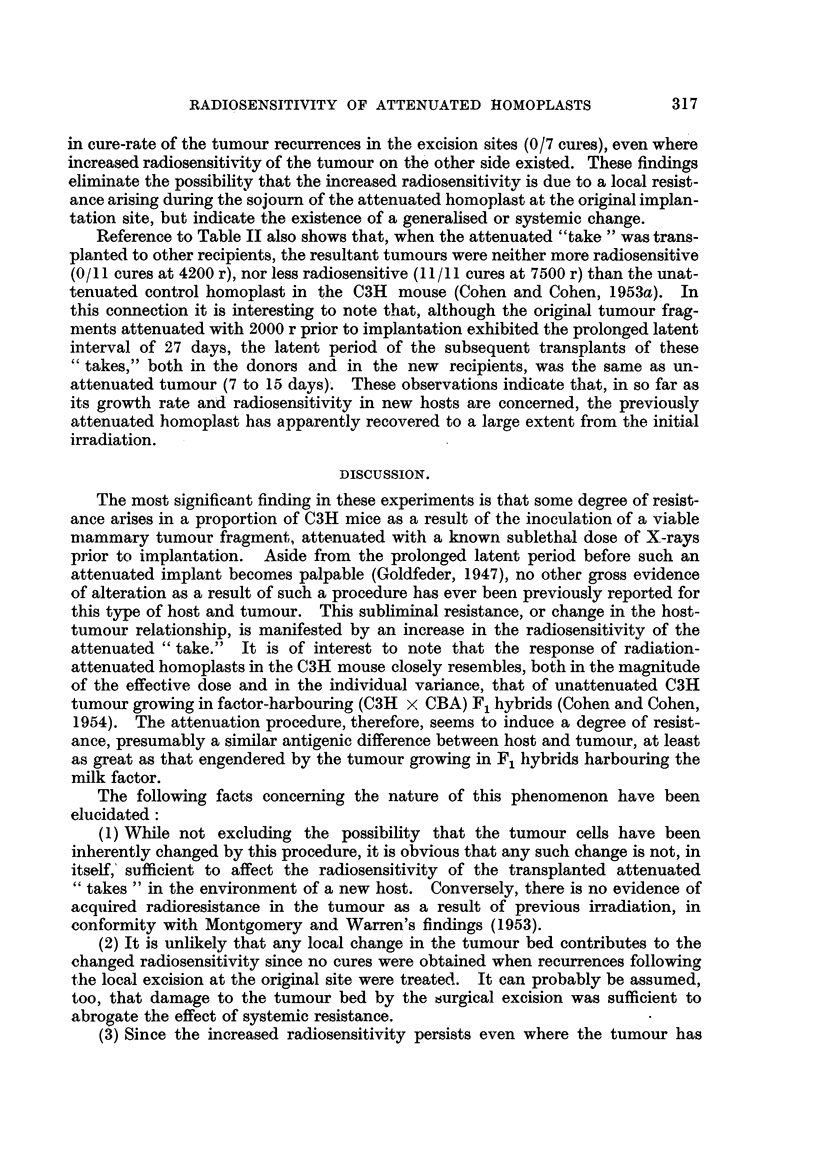

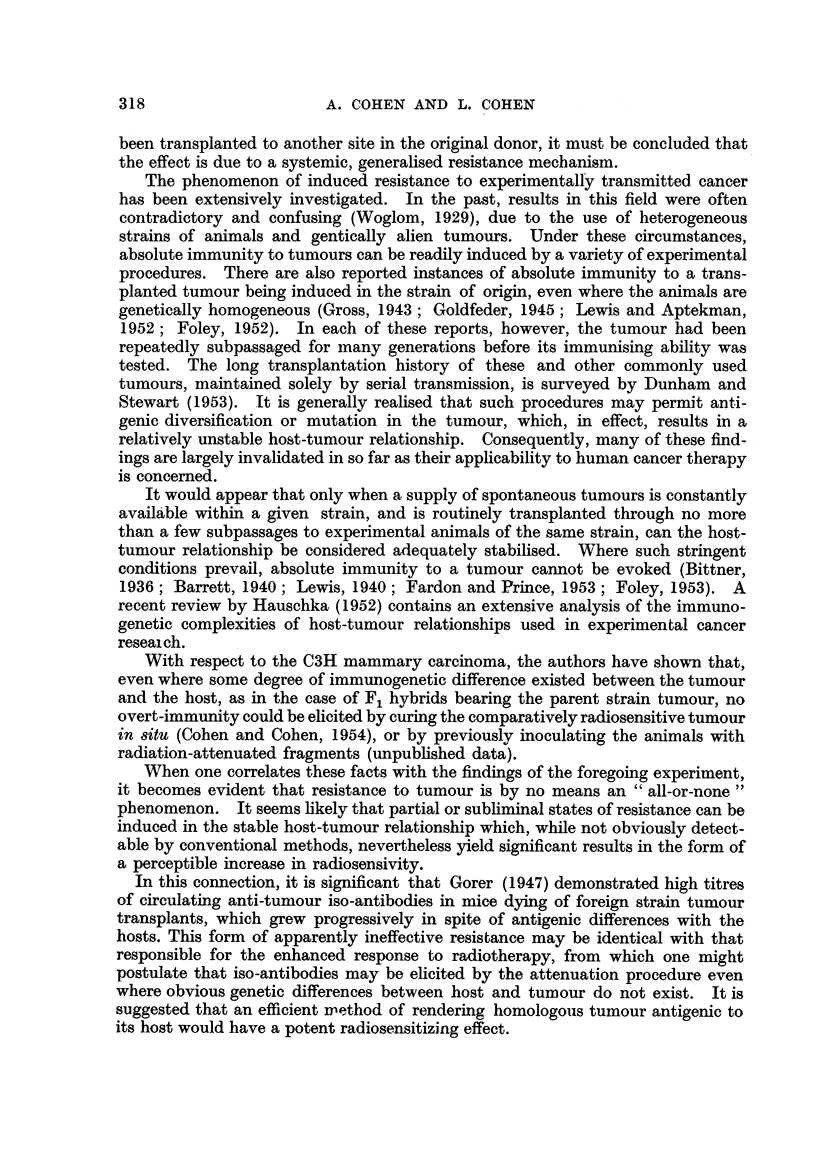

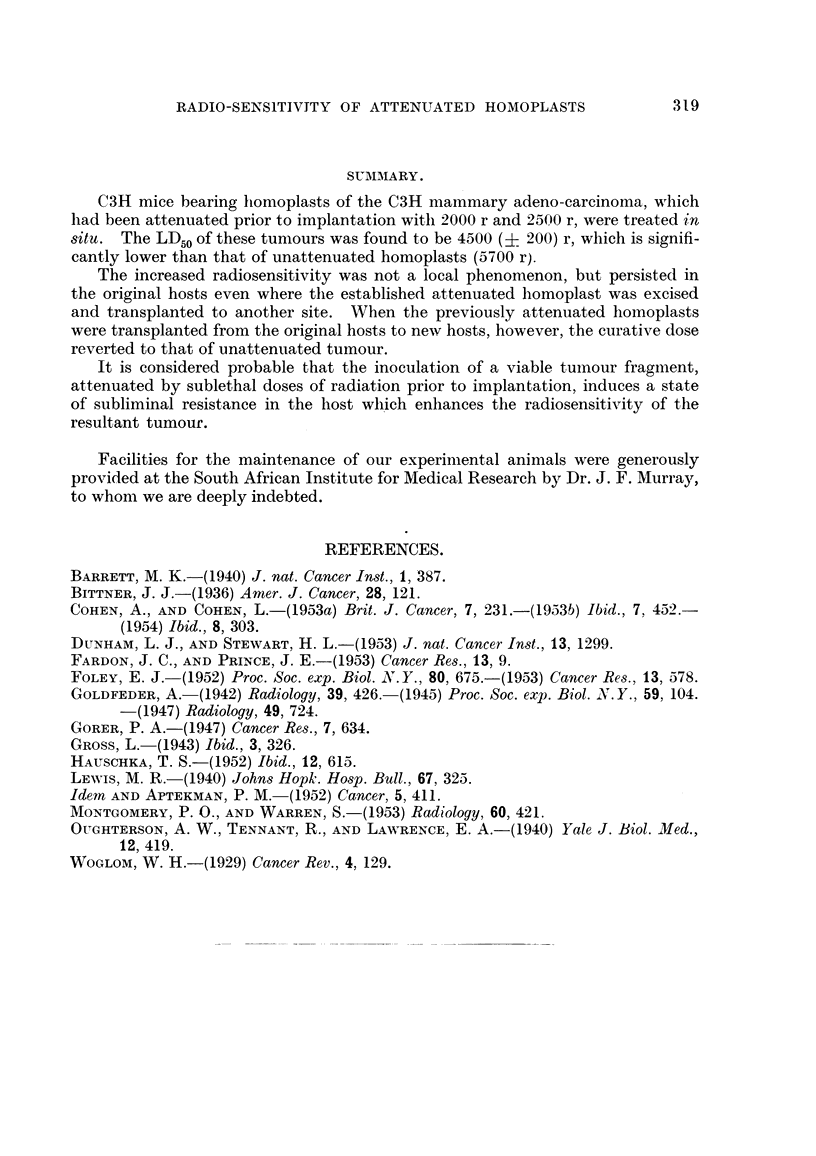


## References

[OCR_00529] DUNHAM L. J., STEWART H. L. (1953). A survey of transplantable and transmissible animal tumors.. J Natl Cancer Inst.

[OCR_00530] FARDON J. C., PRINCE J. E. (1953). An attempt to induce resistance in an inbred strain of mice by ligation of a homologous tumor.. Cancer Res.

[OCR_00532] FOLEY E. J. (1952). Immunity of C3H mice to lymphosarcoma 6-C3H-Ed following regression of the implanted tumor.. Proc Soc Exp Biol Med.

[OCR_00540] HAUSCHKA T. S. (1952). [Immunologic aspects of cancer: a review].. Cancer Res.

[OCR_00543] LEWIS M. R., APTEKMAN P. M. (1952). Atrophy of tumors caused by strangulation and accompanied by development of tumor immunity in rats.. Cancer.

[OCR_00545] MONTGOMERY P. O., WARREN S. (1953). Mechanisms in acquired radioresistance of cancer.. Radiology.

